# Influence of lifestyle factors on breast cancer incidence from mid-life to older age: an Australian longitudinal cohort study

**DOI:** 10.1136/bmjopen-2025-105193

**Published:** 2026-03-29

**Authors:** Md Sohel Rana, M Luke Marinovich, Nehmat Houssami, Dominic Cavenagh, Julie E Byles, Md Mijanur Rahman, Xue Qin Yu

**Affiliations:** 1 The Daffodil Centre, The University of Sydney and Cancer Council NSW, Sydney, New South Wales, Australia; 2 Department of Statistics, Comilla University, Cumilla-3506, Bangladesh; 3 Sydney School of Public Health, Faculty of Medicine and Health, The University of Sydney, Camperdown, New South Wales, Australia; 4 Centre for Women’s Health Research, University of Newcastle, Newcastle, New South Wales, Australia; 5 Hunter Medical Research Institute, New Lambton Heights, New South Wales, Australia; 6 University of Newcastle, Callaghan, New South Wales, Australia; 7 The Collaboration for Cancer Outcomes, Research and Evaluation (CCORE), School of Clinical Medicine, Faculty of Medicine & Health, University of New South Wales, Sydney, New South Wales, Australia

**Keywords:** Breast tumours, PUBLIC HEALTH, ONCOLOGY, Epidemiology, Longitudinal studies

## Abstract

**Abstract:**

**Objective:**

There is limited evidence on the association between lifestyle factors and breast cancer (BC) incidence from Australian longitudinal studies. This study aims to investigate the influence of lifestyle factors on BC incidence over time among Australian women from mid-life to older age.

**Design:**

Longitudinal study.

**Setting:**

Data from the Australian Longitudinal Study on Women’s Health (ALSWH) and linked Australian Cancer Database (ACD).

**Participants:**

12 782 women from the ALSWH 1946–1951 birth cohort linked with the ACD from 1996 to 2019.

**Main outcome and measures:**

Time to the occurrence of BC, accounting for death as a competing event. Body mass index, alcohol consumption, smoking, marital status, oral contraception and hormone replacement therapy were considered as lifestyle factors due to their impact from mid-life to older age.

**Results:**

Among 12 782 women in the cohort, a total of 941 incident BC cases (7.4%) were identified between 1996 and 2019. Time-dependent analysis disclosed that a higher hazard of BC in alcohol drinkers (rarely drinks/low-risk drinkers: Subdistribution HR [sHR]=1.49, 95% CI: 1.33-1.69; risky/high-risk drinkers: sHR=1.36, 95% CI: 1.14-1.62) relative to non-drinkers and those with overweight/obesity (sHR=1.23, 95% CI: 1.14-1.32) relative to underweight/acceptable weight. Results also revealed that non-partnered women (sHR=1.32, 95% CI: 1.12-1.57) had a higher hazard of BC than those with partners. Models were adjusted for lifestyle, reproductive and demographic factors. The probability of cumulative incidence of BC for alcohol drinkers and overweight/obese women steadily increased over time.

**Conclusion:**

This study demonstrated that being non-partnered, overweight/obese and consuming alcohol were associated with increased hazards of BC in women’s mid-life to older age.

**STRENGTHS AND LIMITATIONS OF THIS STUDY**

- This study used longitudinal data from a large prospective cohort linked with the Australian Cancer Database.
- We applied the Fine and Gray subdistribution with time-varying exposure variables to account for competing risk and changes in exposure over time.
- A major limitation of the study is that lifestyle factors in the study were measured through a self-reported questionnaire, and therefore are subject to measurement error and potential misclassifications.
- Some lifestyle factors such as diet pattern and physical activity were not included in the analysis as these variables were inconsistently measured across different survey waves.

## Introduction

 Breast cancer (BC) is one of the most prevalent cancers worldwide, contributing substantial morbidity and mortality among women.^1 2^ Although age and genetic susceptibility are considered the leading risk factors, several modifiable lifestyle factors, such as body weight and alcohol consumption, have attracted increased research attention for their association with BC risk predominantly in women transitioning from mid-life to older age.^3^ The associations between lifestyle factors and BC risk are complex and may differ for different populations, age groups and over time.^4 5^

A range of lifestyle changes occurs as women transition from mid-life to older age, such as menopausal, physiological and shifts in daily practice. Previous research suggests that post-menopausal weight increase is associated with increased incidence of BC.^6 7^ On the other hand, healthy weight management and regular physical activity reduce the risk of BC.^8^,^10^ Both alcohol consumption (even at moderate levels) and smoking (especially when initiated early in life or continued through mid-life) significantly elevate BC risk.^11 12^

As lifestyle factors have multifaceted influences on BC incidence, it is important to delineate their roles over time. Despite numerous prospective and retrospective studies^13^,^19^ examining the association between different lifestyle factors and BC risk, comparatively limited evidence exists from Australian longitudinal studies regarding the direction and strength of these relationships. This is particularly important for understanding BC risk, which develops over a long time during which lifestyle factors may change. Longitudinal studies make it possible to estimate health outcomes due to the effects of cumulative exposure to lifestyle factors. Moreover, longitudinal data permit the assessment of time-dependent factors (eg, body mass index [BMI] and alcohol consumption), which are likely to change with women’s age.^15 20 21^

Understanding how these factors affect BC incidence during this transition period can inform targeted prevention strategies. This study aims to examine the influence of lifestyle on BC incidence over time in a large cohort of Australian women from their mid-life to older age, providing a better understanding of the cumulative and varying impact of lifestyle factors on BC incidence. It is important to examine these associations in the Australian context because of the differences in the lifestyle patterns, healthcare systems and population risk profiles.

## Methods

### Data source and sample

This study utilised data from the 1946–1951 birth cohort of the Australian Longitudinal Study on Women’s Health (ALSWH) with linkage to the Australian Cancer Database (ACD), which contains data about all new cancer cases diagnosed in Australia.^22^ Women in the cohort were invited to participate through random samples using the Medicare Australia Database, which is Australia’s universal health insurance scheme, providing near complete coverage of the population. Estimated response rates were 53–56%.^23^ At baseline (1996), 13 714 women (age: 45–50) completed the self-reported postal questionnaire. These women were first followed up in 1998 and then every 3 years until 2022 (age: 71–76) when the tenth follow-up was completed. However, only follow-up data until 2019 (ninth follow-up) was used, given that the ACD data were not available beyond this year. Retention rates have been high, between 77% and 92%, from the second to ninth survey.^24^ The cohort is broadly representative of the general population of women in this age group, with a slight over-representation of married, Australian-born and tertiary-educated women. Details about the ALSWH survey design, recruitment methods, questionnaires and response rates are available at www.alswh.org.au and have been published elsewhere.^23 25^

The study sample included 12 782 (online supplemental figure A1) women after excluding those who opted out (763) of linking their survey data to administrative data and those with cancer diagnosed before baseline (169).

### Ascertainment of BC cases

The incidence of BC until December 2019 (most recent data available) was identified from the linked ACD using the International Classification of Diseases (ICD-10) code of C50. The ACD comprises nearly complete national information on malignant neoplasms, excluding basal cell carcinomas and squamous cell carcinomas of the skin.^22^ A deterministic linkage based on agreement of common unique identifiers was used for linking cancer information for the ALSWH participants from January 1982 onwards across all Australian states and territories. Details on the linked cancer data are documented elsewhere.^26 27^

### Exposures

Lifestyle factors were the key exposure variables. BMI measured from self-reported height and weight (<18 kg/m^2^=underweight, ≥18 to <25 kg/m^2^=acceptable weight, ≥25 to <30 kg/m^2^=overweight, ≥30 kg/m^2^=obese). Since the prevalence of underweight and obesity is comparatively small, we collapsed BMI into two categories (<25 kg/m^2^=underweight/acceptable weight and ≥25 kg/m^2^=overweight/obese). Data on alcohol consumption were collected based on the question “How often do you usually drink alcohol?” and “On a day when you drink alcohol, how many standard drinks do you usually have?”. According to the National Health and Medical Research Council (NHMRC) guidelines, which align with the WHO guidelines, rarely drinks/low-risk drinkers were defined as those who consume≤10 standard drinks per week or ≤4 on any single day, while risky/high-risk drinkers were those who exceeded these limits. Non-drinkers are those who do not currently consume alcohol. Similarly, smoking status was characterised based on the questions “How often do you currently smoke cigarettes or any tobacco products?” and “In your lifetime, would you have smoked at least 100 cigarettes (or equivalent)?”. The ALSWH derived smoking status variable based on responses from these two questions and labelled (non-smoker, ex-smoker, current smoker). Current use of hormone replacement therapy (HRT) (yes, no), and current user of oral contraception (OC) (yes, no) were also measured. In time-dependent analyses, exposures were measured in each survey until the incidence of BC. Only baseline lifestyle characteristics were used in the non-time-varying analyses.

### Other variables of interest

Sociodemographic variables included: age; area of residence (major cities, inner regional, outer regional, remote and very remote areas)^28^; country of birth (Australia, other English-speaking countries and non-English-speaking countries)^29^; educational qualification (no education/high school, trade/diploma, degree or higher); marital status (partnered and non-partnered); menopausal type (surgical, HRT use, oral contraceptive pill (OCP) use, premenopausal, perimenopausal and postmenopausal); and ability to manage on income (impossible/difficult, not too bad/easy). Reproductive variables were parity (none, one and two or more); age at first full-time pregnancy (11–19 years, 20+ years and not applicable); and age at menarche (<10 years, >10 years and not applicable [participant never had a menstrual period]). However, information on these reproductive variables was only collected in survey 1 or survey 2 with a noticeably higher proportion of missing information (>15% for age at first full-time pregnancy and age at menarche).

Missing values for these variables were filled in if available in the nearest adjacent surveys. However, a small proportion (<5%) of the sociodemographic variables was not filled as there was no available data in adjacent surveys. Complete case analysis was used in all models after filling in missing data from adjacent surveys.

### Statistical analysis

The distribution of the sample by BC cases and non-cases (censored events) was examined based on sociodemographic, reproductive and lifestyle factors using descriptive statistics. We used competing risk survival analysis to determine the association between lifestyle factors and BC incidence over time, accounting for death as a competing event. Time to incident BC was calculated from the return date of the baseline survey to the date of BC diagnosis. Participants were followed up from the return date of the baseline survey until the date of BC diagnosis or death, whichever came first. Those without an event during follow-up were censored at the end of 2019. Fine and Gray competing risk models were used to estimate the subdistribution HRs (sHR) and 95% CIs in two steps. First, using baseline lifestyle characteristics that satisfy the proportional hazard assumption (smoking status, marital status, HRT use, OC use), adjusting for reproductive and demographic characteristics. The cumulative incidence of BC was estimated by applying Fine and Gray’s proportional subdistribution hazard model.^30 31^ A Cox proportional hazards model was also used to evaluate HRs and 95% CI (reported in online supplemental table S3). The cumulative incidence for each event (BC incidence and death) was calculated, accounting for the interdependence of the outcomes. We checked the proportional hazards assumption using the Schoenfeld residuals test.^32^ The assumption held for all variables except for alcohol consumption and BMI.

To address the non-proportionality assumption of alcohol consumption and BMI on BC incidence over time, these variables were separately modelled as time-varying exposures in the second step of the competing risk analysis. To perform the model with time-varying exposures, we prepared our datasets in a counting process style.^33^ Then both unadjusted and adjusted sHR and 95% CIs were estimated using the Fine and Gray competing risk models. We also estimated the cumulative incidence of BC by categories of lifestyle factors from the same model^30^ with time-varying exposures. Analyses were performed in STATA V.18.

## Results

Of 12 782 women in the study cohort, 941 (7.4%) were diagnosed with BC between 1996 and 2019 (table 1). Demographic characteristics were approximately equally distributed in BC cases and those without BC. The majority of women had no education/high school level education in both groups (66.28% vs 64.19%). In both groups, almost three-quarters of women were from major cities and inner regional areas. Women with BC and those without were similar in terms of country of birth, with over three-quarters (75.34% vs 75.77%) born in Australia. The proportion of non-partnered women was slightly higher among BC cases than women without BC (20.40% vs 16.71%). Smoking and alcohol consumption were similarly distributed across groups. Just below one-fifth used HRT and fewer than 10% of women used OC in both the BC cases and non-cases groups (7.23% vs 6.22%). There was a slightly greater proportion of overweight/obesity in the BC cases group (49.95% vs 45.22%). Reproductive variables were also almost equally distributed between the two groups. Details on changes in lifestyle factors over time are presented in online supplemental table S1. In addition, women’s baseline characteristics according to diagnosis with BC, without BC and competing event (death) have been displayed in online supplemental table S2.

**Table 1:** Distribution of women diagnosed with breast cancer (BC) between 1996–2019 and those without BC by baseline (1996) characteristics (n=12 782)

Characteristics	Women without BC n=11 841	Women with BC n=941
Person-years of follow-up time	270 214.48	13 915.03
Age (mean (SD))	47.60 (1.46)	47.62 (1.43)
Education	% (n)	% (n)
No education/high school	66.28 (7848)	64.19 (604)
Trade/diploma	18.83 (2230)	21.15 (199)
Degree or higher	13.88 (1643)	13.71 (129)
Missing	1.01 (120)	0.96 (9)
Place of residence (city)		
Major cities	36.38 (4308)	36.45 (343)
Inner regional	38.27 (4531)	37.51 (353)
Outer regional	20.19 (2391)	21.36 (201)
Remote	3.98 (471)	3.72 (35)
Very remote	1.17 (138)	0.96 (9)
Missing	0.02 (2)	
Country of birth		
Australia	75.34 (8921)	75.77 (713)
Other English speaking	13.26 (1570)	13.18 (124)
non-English speaking	10.15 (1202)	10.20 (96)
Missing	1.25 (148)	0.85 (8)
Marital status		
Partnered	82.81 (9806)	78.96 (743)
Non-partnered	16.71 (1979)	20.40 (192)
Missing	0.47 (56)	0.64 (6)
Menopausal status		
No	63.31 (7496)	61.64 (580)
Yes	35.10 (4156)	37.09 (349)
Missing	1.60 (189)	1.28 (12)
Menopausal type		
Surgical	23.58 (2792)	20.62 (194)
HRT use	9.26 (1097)	10.63 (100)
OCP use	5.51 (652)	6.06 (57)
Premenopausal	33.52 (3969)	33.90 (319)
Perimenopausal	22.57 (2673)	23.49 (221)
Postmenopausal	5.25 (622)	4.99 (47)
Missing	0.30 (36)	0.32 (3)
Age at first pregnancy		
11–19 years	18.47 (2187)	18.38 (173)
20+ years	59.65 (7063)	60.57 (570)
Not applicable	6.35 (752)	5.53 (52)
Missing	15.53 (1839)	15.52 (146)
Age at menarche		
<10 years	4.36 (516)	4.68 (44)
>10 years	79.03 (9358)	79.38 (747)
Not applicable	0.90 (106)	0.53 (5)
Missing	15.72 (1861)	15.41 (145)
Parity		
None	6.14 (727)	5.53 (52)
One	6.29 (745)	7.44 (70)
Two or more	85.35 (10106)	84.91 (799)
Missing	2.22 (263)	2.13 (20)
Ability to manage on income		
Impossible/difficult	43.48 (5149)	42.72 (402)
Not too bad/easy	55.92 (6622)	56.54 (532)
Missing	0.59 (70)	0.74 (7)
Smoking status		
Non-smoker	51.48 (6096)	52.39 (493)
Ex-smoker	27.48 (3254)	26.25 (247)
Current smoker	17.74 (2101)	18.49 (174)
Missing	3.29 (390)	2.87 (27)
Alcohol consumption		
Non-drinker	15.05 (1782)	12.43 (117)
Rarely drinks/low-risk drinks	78.89 (9341)	79.81 (751)
Risky/high-risk drinkers	5.15 (610)	6.80 (64)
Missing	0.91 (108)	0.96 (9)
BMI		
Underweight/acceptable weight	50.15 (5938)	46.65 (439)
Overweight/obese	45.22 (5355)	49.95 (470)
Missing	4.63 (548)	3.40 (32)
OC use		
No	93.51 (11073)	92.56 (871)
Yes	6.22 (737)	7.23 (68)
Missing	0.26 (31)	0.21 (2)
HRT use		
No	80.80 (9567)	79.91 (752)
Yes	18.98 (2248)	19.55 (184)
Missing	0.22 (26)	0.53 (5)

According to International Classification of Diseases-10 code C50, there were 950 BC cases. However, for nine cases, the date of diagnosis is missing in the Australian Cancer Database, and as our outcome of interest is time to the occurrence of BC, we considered these nine cases as censored.

BMI, body mass index; HRT, hormone replacement therapy; OC, oral contraception; OCP, oral contraceptive pill; SD, standard deviation.

Table 2 presents both the adjusted and unadjusted sHR with corresponding 95% CI. The results reveal that alcohol consumption increased the hazard of BC incidence irrespective of drinking level (rarely drinks/low-risk drinkers: adjusted sHR=1.49, 95% CI: 1.33-1.69, risky/high-risk drinkers: adjusted sHR=1.36, 95% CI: 1.14-1.62). Overweight/obese women had significantly higher risks of BC incidence compared with underweight/acceptable weight (adjusted sHR=1.23, 95% CI: 1.14-1.32). Non-partnered women had a 32% higher risk of BC incidence than partnered women (adjusted sHR=1.32, 95% CI: 1.12-1.57). Other lifestyle factors, such as smoking status, OC and HRT, did not show significant associations with BC incidence. The reproductive life variables (parity, age at first full-time pregnancy and age at menarche) were not significantly associated with BC incidence (online supplemental table S3).

**Table 2:** The HR and 95% CI of lifestyle factors and breast cancer incidence among Australian women born 1946–1951 (n=12 782)

Lifestyle factors	Unadjusted sHR (95% CI)	Adjusted sHR (95% CI)
Smoking status		
Non-smoker	Ref	Ref
Ex-smoker	0.94 (0.81 to 1.10)	0.92 (0.78 to 1.07)
Current smoker	1.03 (0.86 to 1.22)	0.96 (0.80 to 1.16)
Cox-proportional global test	p=0.32	p=0.25
Alcohol consumption*		
Non-drinker	Ref	Ref
Rarely drinks/low-risk drinkers	1.56 (1.40 to 1.74)	1.49 (1.33 to 1.69)
Risky/high-risk drinkers	1.48 (1.25 to 1.76)	1.36 (1.14 to 1.62)
Cox-proportional global test	p<0.01	p<0.05
BMI*		
Underweight/acceptable weight	Ref	Ref
Overweight/obese	1.09 (1.02 to 1.17)	1.23 (1.14 to 1.32)
Cox-proportional global test	p<0.05	p<0.01
Marital status		
Partnered	Ref	Ref
Non-partnered	1.27 (1.08 to 1.49)	1.32 (1.12 to 1.57)
Cox-proportional global test	p=0.37	p=0.29
OC use		
No	Ref	Ref
Yes	1.16 (0.91 to 1.49)	1.15 (0.89 to 1.49)
Cox-proportional global test	p=0.91	p=0.76
HRT use		
No	Ref	Ref
Yes	1.04 (0.88 to 1.22)	1.05 (0.89 to 1.24)
Cox-proportional global test	p=0.92	p=0.75

Multivariate model includes all the lifestyle factors and is adjusted for age, income, area of residence, country of birth, education, parity, age at first birth and age at menarche.

Cox-proportional global test p<0.05 represents statistical significance of violation of proportionality assumptions.

* Modelled by taking into account their time-varying effects as they violated the proportionality assumptions. In time-varying modelling, the sample size n=12 620 (online supplemental figure A2) because 162 women were participated only on the baseline survey in 1996. Therefore, there was no chance of time-varying exposure for those women.

BMI, body mass index; HRT, hormone replacement therapy; OC, oral contraception; Ref, reference category; sHR, subdistribution HR.

**Figure 1:**
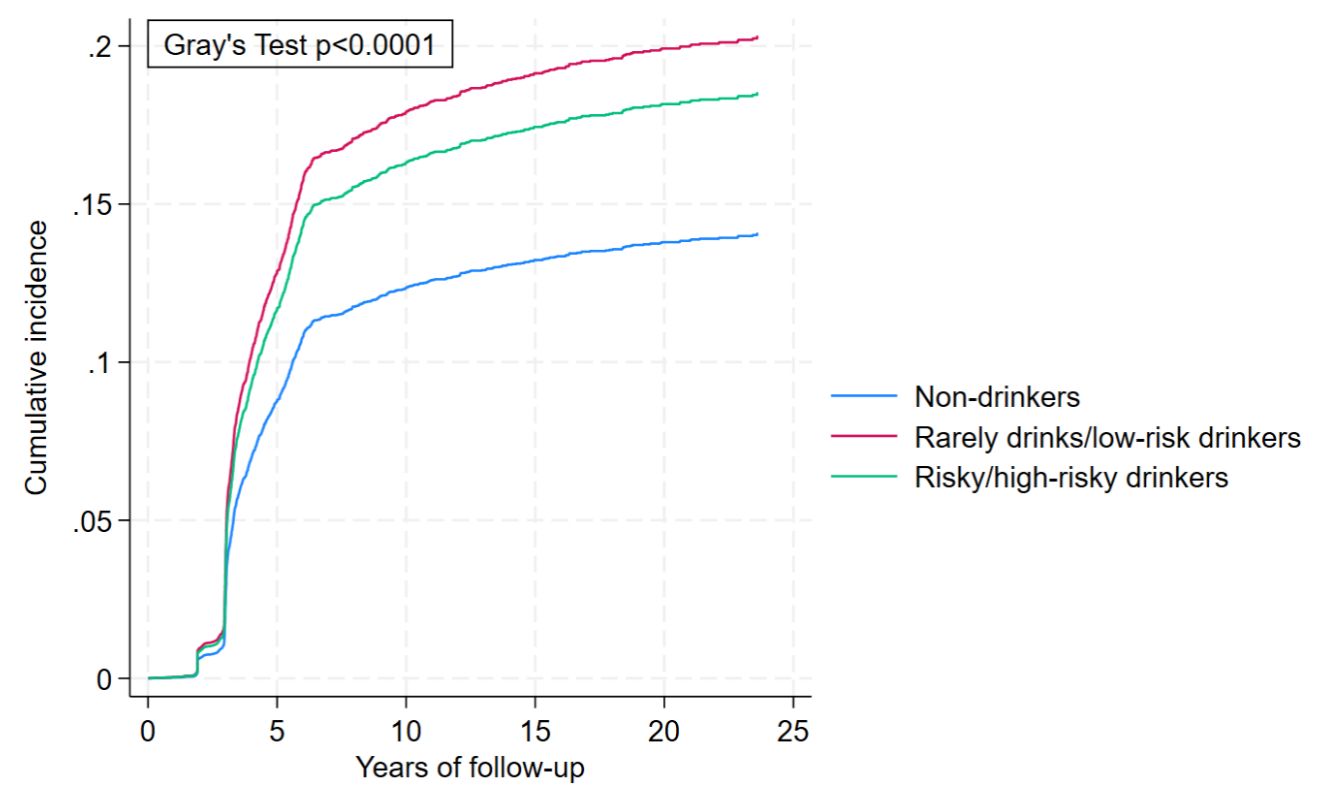
Cumulative incidence of breast cancer incidence by level of alcohol consumption considering its time-varying effects, accounting for death as a competing event.

The cumulative incidence curve (figure 1) showed that alcohol consumption at any level was associated with increased BC incidence, as the probability of rarely drinks/low-risk drinkers was significantly higher among risky/high-risk drinkers higher than among non-drinkers over time with p<0.01. Therefore, alcohol consumption is a significant risk factor for BC irrespective of the level of alcohol drinking. The probability of the cumulative incidence began differing after 5 years of follow-up (0.13 vs 0.12 vs 0.09). After 23 years of follow-up, the probability of cumulative incidence for the former two groups steadily increased (0.20 and 0.18) and established an increased pattern over time.

**Figure 2:**
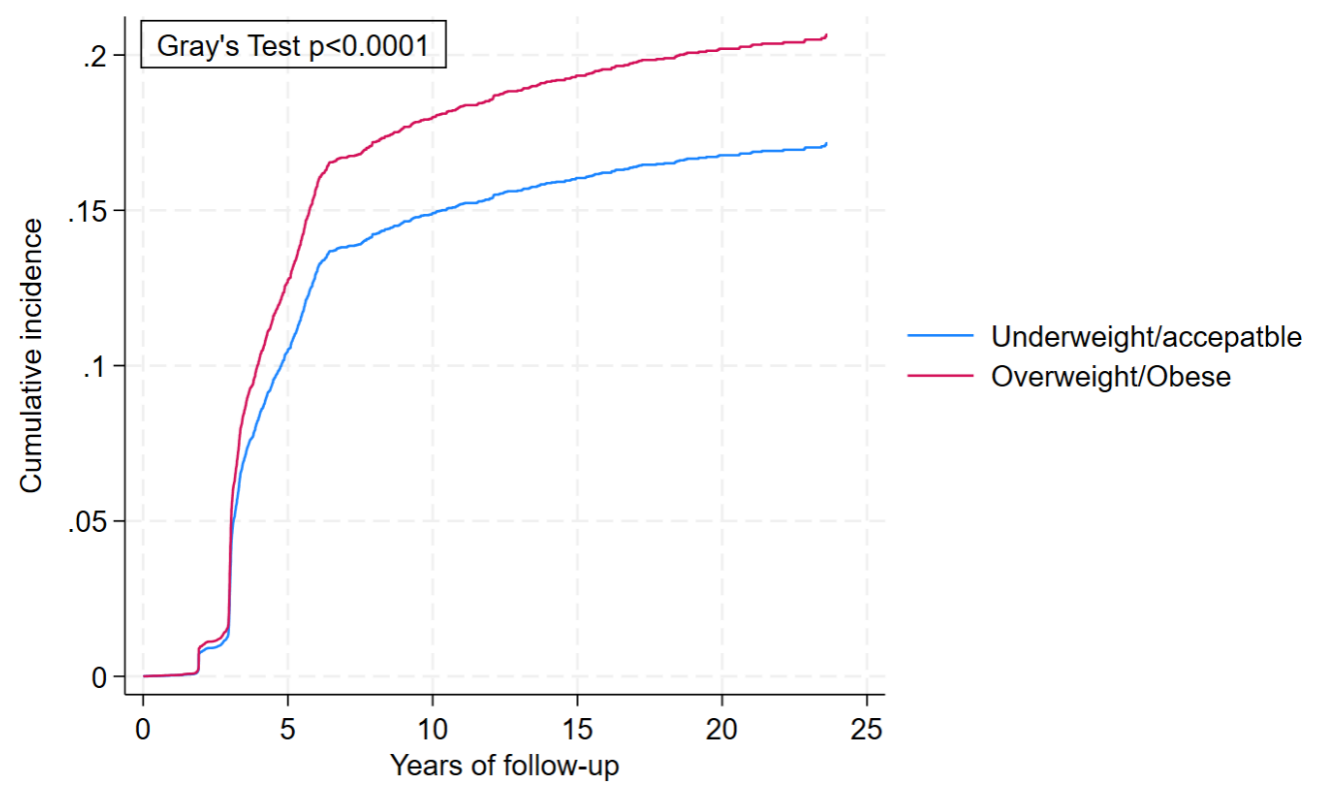
Cumulative incidence of breast cancer incidence by body mass index categories considering time-varying effects, accounting for death as a competing event.

The cumulative incidence of BC incidence by BMI categories revealed that the probability of women who were overweight/obese was significantly higher than that of underweight/acceptable weight women with p<0.01 (figure 2). The probability of cumulative incidence in the former category increased more sharply than the latter category over time, and at 20 years of follow-up, the chances were 0.20 and 0.17, respectively.

## Discussion

This study investigated the association between lifestyle factors and BC incidence in a large cohort of Australian women from mid-life to older age, integrating sustained follow-up and using appropriate analytic methods. Our study found that alcohol consumption, being overweight/obese and not having a partner significantly increased the hazard of BC incidence in this cohort. It was also evident that alcohol consumption and being overweight/obese steadily increased the probability of BC incidence over time.

Alcohol consumption exhibited a significant positive association with BC incidence, with consideration of time-dependent impacts. It is well documented that alcohol consumption is associated with increased risk of BC incidence; alcohol increases oestrogen circulation and DNA destruction, both of which influence the progression of BC.^34^,^38^ Over time, the association remained significant irrespective of the drinking patterns. Previous research also reported that any amount of alcohol consumption increases BC risk and longer duration of moderate alcohol consumption substantially escalates the incidence of BC.^39^,^41^

Our study also revealed a significantly higher BC incidence among overweight/obese women compared with underweight/acceptable weight women, which is consistent with previous studies.^42 43^ Previous literature documented that longer duration of being overweight/obese^44 45^ increases risk of BC. For postmenopausal BC, obesity is a well-known risk factor.^6 46 47^ Additional body fat promotes excessive oestrogen production in adipose tissue, chronic inflammation and insulin resistance, all of which contribute to BC progression.^48 49^ Our study highlights the significance of healthy weight management as a modifiable lifestyle factor from midlife to older age for preventing BC.^50^

Our study also found that non-partnered women had an increased hazard of BC incidence. Earlier studies have also reported that non-partnered (vs partnered) women were at higher hazard of BC development^51^ and suggested that partnered women might enjoy social and emotional supports which have protective health effects, including for cancer.^52 53^ Women who live with a partner may be more health conscious and seek better healthcare, maintain healthier lifestyle behaviours and regularly uphold preventive care, with consequently decreased BC incidence. On the other hand, non-partnered women may be more likely to experience stress and social isolation, which potentially contribute to long-term inflammation and impaired autoimmunity, which are interlinked with oncogenesis.^54 55^

We found a statistically non-significant association between smoking and BC incidence, which is in line with previous studies that reported a null association, especially in postmenopausal women.^56 57^ In our study, follow-up began when women were aged between 45 and 50 years, corresponding closely to the typical onset of menopause (45–55 years). Other studies show inconsistent findings on the relationship between smoking and BC, with some showing positive associations depending on the duration and intensity of smoking and premenopausal status.^1258^,^60^ The inconclusive relationship may reflect the complexity of smoking as a risk factor, with other cofactors such as hormonal effects, duration, intensity and starting age of smoking potentially modifying the association.^57 61^

Our study found that HRT and OC users had higher hazards of BC incidence, but these associations are not statistically significant. These findings are consistent with meta-analytic findings of small effects of long-term combined oestrogen-progestin therapy on BC incidence depending on the menopause.^62^ Similarly, long-term OC use among young women is associated with a moderate increase in BC incidence; however, risk reduces after stopping use.^63^ Confounding by other reproductive factors and relatively shorter duration of OC use may have contributed to non-significant associations in our study.

This study has several strengths, including the use of a large population sample from a well-defined prospective longitudinal study (ALSWH) with a high retention rate. We verified BC cases and deaths from the ACD, which reduces the chance of misclassification. We also accounted for longitudinal changes in lifestyle factors (BMI, alcohol), which may follow a trajectory^64^,^66^ in assessing their cumulative effects on BC incidence. Our analysis considered competing events (death) to provide a more robust estimate of BC incidence in a population transitioning from mid-life to older age. We adjusted our models for potential reproductive and demographic variables that warranted less confounding. Our study also had some limitations. Lifestyle factors in the ALSWH were subjective, so there is the potential for misclassifications; these misclassifications are likely to be non-differential and may bias the finding towards the null.^67^ We did not include some other lifestyle factors such as diet pattern and physical activity in the analysis as these variables were inconsistently measured across different survey waves. Potential interaction effects were not assessed in our study due to the complexity of modelling time-varying interactions, which may limit the ability to determine whether the observed associations differed across subgroups. We did not account for the level of smoking (eg, number of cigarettes per day, pack-years) due to inconsistency across survey waves and the first pregnancy after age 30, as it is not asked at the baseline survey. In addition, women with missing information may be likely to have poorer lifestyle factors and higher hazards of BC.

## Conclusion

The study findings on significantly increased hazard of BC incidence associated with higher BMI, alcohol consumption, being non-partnered emphasises the importance of healthy weight management, cessation of alcohol consumption and attention to psychosocial requirements of non-partnered women to prevent BC incidence. The cumulative impacts of alcohol and BMI on BC incidence in time-dependent analyses highlight the importance of long-term intervention strategies to modify BC risks. In addition, we found that the association between smoking, OC, HRT and BC incidence was not statistically significant. Future research focus should be on understanding the mechanisms by which these lifestyle factors (alcohol and BMI) influence BC incidence over time, especially during the transition period of women from their mid-life to older age, and the implications for intervention strategies.

## Supplementary material

10.1136/bmjopen-2025-105193 online supplemental file 1

## Data Availability

Data may be obtained from a third party and are not publicly available.
