# Supplementary material for: Influence of lifestyle factors on breast cancer incidence from mid-life to older age: an Australian longitudinal cohort study
Source: BMJ Open. 2026 Mar 29;16(3):e105193. doi: 10.1136/bmjopen-2025-105193 (PMC13034360; doi:10.1136/bmjopen-2025-105193)
Supplement: online supplemental file 1 [file bmjopen-16-3-s001.docx]

# Supplementary

The following figure displays the sample included in the analyses of the study.

Australian Longitudinal Study on Women’s Health (ALSWH) 1946-1951 cohort

**(N=13714)**

**Censored**

Women without BC up to 31 December 2019

**(N=10779)**

Opted out to link their data to the ACD

**(N=763)**

**Competing event**

Died during the follow-up **(N=1062)**

Women without breast cancer followed up until

31 December 2019

**(N=12782)**

Diagnosed with BC before the baseline survey in 1996 **(N=169)**

Women in ALSWH data linked to the ACD

**(N=12951)**

**Event of interest**

Diagnosed with BC during the follow-up, 31 December 2019

**(N=941)**

Figure A1: Flowchart of included women in the study from 1946-1951 cohort of ALSWH

The following figure presents the inclusion criteria and sample used in the time-varying analyses.

Australian Longitudinal Study on Women’s Health (ALSWH) 1946-1951 cohort

**N=13714**

Opted out to link their data to the ACD

**N=763**

Died during the follow-up **N=1062**

Women diagnosed with BC during the follow-up time (31 December 2019)

**N=941**

Excluded who participated only 1^st^ survey and not died/diagnosed/withdrawn

**N=162**

Women in ALSWH data linked to the ACD

**N=12951**

Diagnosed with breast cancer before the baseline survey in 1996

**N=169**

Women without breast cancer followed up until

31 December 2019

**N=12782**

Women alieved without BC up to 31 December 2019

**N=10617**

Women included in the study

**N=12620**

Figure A2: Flowchart of included women in the time varying covariates study from 1946-1951 cohort of ALSWH

Table S1: Characteristics of ten survey rounds of mid- aged cohort (1946-1951) of ALSWH

| Characteristics | Wave 1  (N=12782) | Wave 2  (N=11518) | Wave 3  (N=10492) | Wave 4  (N=10184) | Wave 5  (N=9916) | Wave 6  (N=9317) | Wave 7  (N=8571) | Wave 8  (N=8129) | Wave 9  (N=7516) | Wave 10  (N=6768) |
| --- | --- | --- | --- | --- | --- | --- | --- | --- | --- | --- |
|  | % (N) | % (N) | % (N) | % (N) | % (N) | % (N) | % (N) | % (N) | % (N) |  |
| BMI |  |  |  |  |  |  |  |  |  |  |
| Underweight | 1.66 (212) | 1.26 (145) | 1.28 (134) | 1.01 (103) | 1.08 (107) | 1.21 (113) | 1.12 (96) | 1.39 (113) | 1.37 (103) | 1.8 (122) |
| Normal weight | 48.23 (6165) | 41.09 (4733) | 39.68 (4163) | 36.91 (3759) | 36.49 (3618) | 34.8 (3242) | 33.49 (2870) | 32.59 (2649) | 31.45 (2364) | 34.04 (2304) |
| Overweight | 27.42 (3505) | 27.44 (3161) | 30.22 (3171) | 31.31 (3189) | 32.9 (3262) | 33.57 (3128) | 32.6 (2794) | 31.82 (2587) | 29.51 (2218) | 33.14 (2243) |
| Obese | 18.15 (2320) | 18.05 (2079) | 22.27 (2337) | 24.81 (2527) | 26.9 (2667) | 28.01 (2610) | 29.14 (2498) | 29.17 (2371) | 25.28 (1900) | 28.34 (1918) |
| Missing | 4.54 (580) | 12.15 (1400) | 6.55 (687) | 5.95 (606) | 2.64 (262) | 2.4 (224) | 3.65 (313) | 5.03 (409) | 12.39 (931) | 2.67 (181) |
| Physical Activity |  |  |  |  |  |  |  |  |  |  |
| Nil/Sedentary |  | 16.36 (1884) | 16.38 (1719) | 15.01 (1529) | 14.68 (1456) | 15.36 (1431) | 16.38 (1404) | 16.04 (1304) | 16.52 (1242) | 20.04 (1356) |
| Low |  | 26.05 (3001) | 31.93 (3350) | 23.16 (2359) | 20.78 (2061) | 21.5 (2003) | 19.88 (1704) | 18.83 (1531) | 23.98 (1802) | 22.53 (1525) |
| Moderate |  | 20.85 (2401) | 20.26 (2126) | 22 (2240) | 22.2 (2201) | 20.45 (1905) | 20.56 (1762) | 20.33 (1653) | 20.93 (1573) | 19.65 (1330) |
| High |  | 27.93 (3217) | 26.9 (2822) | 33.16 (3377) | 36.35 (3604) | 36.21 (3374) | 39.59 (3393) | 40.62 (3302) | 34.18 (2569) | 32.73 (2215) |
| Missing |  | 8.81 (1015) | 4.53 (475) | 6.67 (679) | 5.99 (594) | 6.48 (604) | 3.59 (308) | 4.17 (339) | 4.39 (330) | 5.05 (342) |
| Smoking |  |  |  |  |  |  |  |  |  |  |
| Non-smoker | 51.55 (6589) | 52.96 (6100) | 61 (6400) | 59.16 (6025) | 59.93 (5943) | 60.86 (5670) | 62.41 (5349) | 62.66 (5094) | 64.12 (4819) | 64.91 (4393) |
| Ex. smoker | 27.39 (3501) | 24.86 (2863) | 24.18 (2537) | 27.13 (2763) | 28.83 (2859) | 30.06 (2801) | 30.17 (2586) | 31.09 (2527) | 30.39 (2284) | 30.35 (2054) |
| Current Smoker | 17.8 (2275) | 15.93 (1835) | 14.4 (1511) | 13.38 (1363) | 10.84 (1075) | 8.77 (817) | 6.57 (563) | 5.25 (427) | 4.16 (313) | 3.44 (233) |
| Missing | 3.26 (417) | 6.25 (720) | 0.42 (44) | 0.32 (33) | 0.39 (39) | 0.31 (29) | 0.85 (73) | 1 (81) | 1.33 (100) | 1.3 (88) |
| OC use |  |  |  |  |  |  |  |  |  |  |
| No | 93.44 (11944) | 89.67 (10328) | 97.42 (10221) | 99.3 (10113) | 99.5 (9866) | 99.59 (9279) |  |  |  |  |
| Yes | 6.3 (805) | 4.15 (478) | 2.56 (269) | 0.7 (71) | 0.14 (14) | 0.1 (9) |  |  |  |  |
| Missing | 0.26 (33) | 6.18 (712) | 0.02 (2) |  | 0.36 (36) | 0.31 (29) |  |  |  |  |
| HRT use |  |  |  |  |  |  |  |  |  |  |
| No | 80.73 (10319) | 70.99 (8177) | 66.59 (6987) | 77.86 (7929) | 44.56 (4419) | 87.55 (8157) | 90.35 (7744) | 91.2 (7414) | 91.95 (6911) | 91.19 (6172) |
| Yes | 19.03 (2432) | 22.19 (2556) | 33.39 (3503) | 22.14 (2255) | 54.75 (5429) | 11.9 (1109) | 9.37 (803) | 8.54 (694) | 7.08 (532) | 5.95 (403) |
| Missing | 0.24 (31) | 6.82 (785) | 0.02 (2) |  | 0.69 (68) | 0.55 (51) | 0.28 (24) | 0.26 (21) | 0.97 (73) | 2.85 (193) |
| Alcohol |  |  |  |  |  |  |  |  |  |  |
| Low risk | 47.97 (6131) | 49.36 (5685) |  | 53.85 (5484) | 56.47 (5600) | 55.28 (5150) | 53.84 (4615) | 53.76 (4370) | 67.72 (5090) | 46.69 (3160) |
| Non-drinker | 14.86 (1899) | 12.4 (1428) |  | 14.94 (1521) | 13.75 (1363) | 14.22 (1325) | 16.15 (1384) | 16.95 (1378) | 7.74 (582) | 19.7 (1333) |
| Rarely drinks | 30.99 (3961) | 26.65 (3069) |  | 23.5 (2393) | 22.54 (2235) | 23.02 (2145) | 22.83 (1957) | 21.54 (1751) | 11.59 (871) | 23.89 (1617) |
| Risky drinkers | 4.44 (567) | 4.59 (529) |  | 5.71 (581) | 5.46 (541) | 6.03 (562) | 5.33 (457) | 5.43 (441) | 4.23 (318) | 4.08 (276) |
| High risk drinkers | 0.84 (107) | 0.76 (87) |  | 0.93 (95) | 0.81 (80) | 0.65 (61) | 0.54 (46) | 0.6 (49) | 0.19 (14) | 0.3 (20) |
| Missing | 0.92 (117) | 6.25 (720) |  | 1.08 (110) | 0.98 (97) | 0.79 (74) | 1.31 (112) | 1.72 (140) | 8.53 (641) | 5.35 (362) |
| Menopausal status |  |  |  |  |  |  |  |  |  |  |
| Surgical | 23.36 (2986) | 25.92 (2985) | 28.93 (3035) | 31.31 (3189) | 33.09 (3281) | 33.59 (3130) |  |  |  |  |
| HRT | 9.36 (1197) | 11.3 (1301) | 17.08 (1792) | 9.82 (1000) | 6.47 (642) | 3.78 (352) |  |  |  |  |
| OCP | 5.55 (709) | 3.88 (447) | 2.1 (220) | 0.47 (48) | 0.08 (8) |  |  |  |  |  |
| Pre | 33.55 (4288) | 22.77 (2623) | 9.04 (948) | 2.04 (208) | 0.17 (17) | 0.01 (1) |  |  |  |  |
| Peri | 22.64 (2894) | 24.31 (2800) | 17.89 (1877) | 9.65 (983) | 1.99 (197) | 0.2 (19) |  |  |  |  |
| Post | 5.23 (669) | 11.36 (1308) | 24.34 (2554) | 46.33 (4718) | 58.01 (5752) | 60.34 (5622) |  |  |  |  |
| Missing | 0.31 (39) | 0.47 (54) | 0.63 (66) | 0.37 (38) | 0.19 (19) | 2.07 (193) |  |  |  |  |
| Education |  |  |  |  |  |  |  |  |  |  |
| No education/high school | 17.85 (2281) | 16.79 (1934) | 16.31 (1711) | 16.06 (1636) | 15.93 (1580) | 14.96 (1394) | 14.02 (1202) | 13.68 (1112) | 12.89 (969) | 12.25 (829) |
| Trade/diploma | 67.28 (8600) | 67.93 (7824) | 68.22 (7158) | 68.21 (6946) | 68.21 (6764) | 68.75 (6405) | 68.78 (5895) | 68.79 (5592) | 68.79 (5170) | 68.65 (4646) |
| University or higher | 13.86 (1772) | 14.36 (1654) | 14.69 (1541) | 14.92 (1519) | 15.06 (1493) | 15.51 (1445) | 16.45 (1410) | 16.82 (1367) | 17.64 (1326) | 18.44 (1248) |
| Missing | 1.01 (129) | 0.92 (106) | 0.78 (82) | 0.82 (83) | 0.8 (79) | 0.78 (73) | 0.75 (64) | 0.71 (58) | 0.68 (51) | 0.66 (45) |
| Place of residence |  |  |  |  |  |  |  |  |  |  |
| Major cities | 36.39 (4651) | 35.88 (4133) | 33.76 (3542) | 37.49 (3818) | 37.3 (3699) | 37.51 (3495) | 38.19 (3273) | 38.73 (3148) | 38.89 (2923) | 38.76 (2623) |
| Inner regional | 38.21 (4884) | 38.63 (4449) | 40.68 (4268) | 39.17 (3989) | 39.25 (3892) | 40.29 (3754) | 39.8 (3411) | 40.46 (3289) | 41.18 (3095) | 39.67 (2685) |
| Outer regional | 20.28 (2592) | 20.45 (2356) | 20.56 (2157) | 19.4 (1976) | 19.43 (1927) | 18.62 (1735) | 18.84 (1615) | 17.85 (1451) | 17.14 (1288) | 15.69 (1062) |
| Remote | 3.96 (506) | 3.95 (455) | 3.46 (363) | 3 (306) | 2.9 (288) | 2.64 (246) | 2.23 (191) | 2.04 (166) | 1.97 (148) | 1.37 (93) |
| Very remote | 1.15 (147) | 1.08 (124) | 0.89 (93) | 0.82 (83) | 0.84 (83) | 0.77 (72) | 0.63 (54) | 0.44 (36) | 0.39 (29) | 0.46 (31) |
| Missing | 0.02 (2) | 0.01 (1) | 0.66 (69) | 0.12 (12) | 0.27 (27) | 0.16 (15) | 0.32 (27) | 0.48 (39) | 0.44 (33) | 4.05 (274) |
| Marital Status |  |  |  |  |  |  |  |  |  |  |
| Partnered | 82.53 (10549) | 82.09 (9455) | 81.13 (8512) | 79.46 (8092) | 77.81 (7716) | 76.29 (7108) | 73.97 (6340) | 71.36 (5801) | 67.62 (5082) | 65.23 (4415) |
| Non-partnered | 16.98 (2171) | 17.24 (1986) | 18.4 (1931) | 19.16 (1951) | 21.36 (2118) | 22.96 (2139) | 25 (2143) | 27.64 (2247) | 30.53 (2295) | 33.24 (2250) |
| Missing | 0.49 (62) | 0.67 (77) | 0.47 (49) | 1.38 (141) | 0.83 (82) | 0.75 (70) | 1.03 (88) | 1 (81) | 1.85 (139) | 1.52 (103) |

Table S2: Baseline characteristics of study participants in 1996, according to the first event, competing event or censoring during follow-up (N=12782)

| Characteristics | No event (without BC cases)  N=10779 | Event (incidence of BC cases)  N=941 | Competing event (Death)  N=1062 |
| --- | --- | --- | --- |
| Age [Mean(SD)] | 47.55 (1.46) | 47.62 (1.43) | 47.77 (1.46) |
| Education | % (N) | % (N) | % (N) |
| No education/high school | 65.57 (7068) | 64.19 (604) | 73.45 (780) |
| Trade/Diploma | 18.97 (2045) | 21.15 (199) | 17.42 (185) |
| University or Higher | 14.49 (1562) | 13.71 (129) | 7.63 (81) |
| Missing | 0.96 (104) | 0.96 (9) | 1.51 (16) |
| Place of residence |  |  |  |
| Major cities | 36.72 (3958) | 36.45 (343) | 32.96 (350) |
| Inner regional | 38.24 (4122) | 37.51 (353) | 38.51 (409) |
| Outer regional | 20.04 (2160) | 21.36 (201) | 21.75 (231) |
| Remote | 3.84 (414) | 3.72 (35) | 5.37 (57) |
| Very remote | 1.14 (123) | 0.96 (9) | 1.41 (15) |
| Missing | 0.02 (2) | 36.45 (343) | 32.96 (350) |
| Country of birth |  |  |  |
| Australia | 75.01 (8085) | 75.77 (713) | 78.72 (836) |
| Other English speaking | 13.4 (1444) | 13.18 (124) | 11.86 (126) |
| Europe | 6.61 (712) | 6.48 (61) | 4.99 (53) |
| Asia | 2.81 (303) | 2.23 (21) | 1.6 (17) |
| Other | 0.98 (106) | 1.49 (14) | 1.04 (11) |
| Missing | 1.20 (129) | 0.85 (8) | 1.79 (19) |
| Marital status |  |  |  |
| Partnered | 83.42 (8992) | 78.96 (743) | 76.65 (814) |
| Non-partnered | 16.11 (1736) | 20.4 (192) | 22.88 (243) |
| Missing | 0.47 (51) | 0.64 (6) | 0.47 (5) |
| Menopausal status |  |  |  |
| No | 63.85 (6882) | 61.64 (580) | 57.82 (614) |
| Yes | 34.56 (3725) | 37.09 (349) | 40.58 (431) |
| Missing | 1.6 (172) | 1.28 (12) | 1.6 (17) |
| Menopausal type |  |  |  |
| Surgical | 23.12 (2492) | 20.62 (194) | 28.25 (300) |
| HRT use | 8.89 (958) | 10.63 (100) | 13.09 (139) |
| OCP use | 5.58 (601) | 6.06 (57) | 4.8 (51) |
| Pre-menopausal | 34.37 (3705) | 33.9 (319) | 24.86 (264) |
| Peri-menopausal | 22.66 (2443) | 23.49 (221) | 21.66 (230) |
| Post-menopausal | 5.11 (551) | 4.99 (47) | 6.69 (71) |
| Missing | 0.27 (29) | 0.32 (3) | 0.66 (7) |
| Ability to manage on income |  |  |  |
| Impossible-difficult | 42.61 (4593) | 42.72 (402) | 52.35 (556) |
| Not too bad-easy | 56.81 (6124) | 56.54 (532) | 46.89 (498) |
| Missing | 0.58 (62) | 0.74 (7) | 0.75 (8) |
| Age at first pregnancy |  |  |  |
| 11-19 Years | 18.29 (1971) | 18.38 (173) | 20.34 (216) |
| 20+ Years | 60.84 (6558) | 60.57 (570) | 47.55 (505) |
| Not applicable | 6.13 (661) | 5.53 (52) | 8.57 (91) |
| Missing | 14.74 (1589) | 15.52 (146) | 23.54 (250) |
| Age at menarche |  |  |  |
| <10 Years | 4.27 (460) | 4.68 (44) | 5.27 (56) |
| >10 Years | 79.97 (8620) | 79.38 (747) | 69.49 (738) |
| Not applicable | 0.85 (92) | 0.53 (5) | 1.32 (14) |
| Missing | 14.91 (1607) | 15.41 (145) | 23.92 (254) |
| Parity |  |  |  |
| None | 5.89 (635) | 5.53 (52) | 8.66 (92) |
| One | 6.06 (653) | 7.44 (70) | 8.66 (92) |
| Two or more | 85.94 (9263) | 84.91 (799) | 79.38 (843) |
| Missing | 2.12 (228) | 2.13 (20) | 3.30 (35) |
| Smoking Status |  |  |  |
| Non-smoker | 52.94 (5706) | 52.39 (493) | 36.72 (390) |
| Ex-smoker | 27.81 (2998) | 26.25 (247) | 24.11 (256) |
| Current smoker | 16.02 (1727) | 18.49 (174) | 35.22 (374) |
| Missing | 3.23 (348) | 2.87 (27) | 3.95 (42) |
| Alcohol consumption |  |  |  |
| Non-drinker | 14.68 (1582) | 12.43 (117) | 18.83 (200) |
| Rarely drinks/low-risk drinkers | 79.61 (8581) | 79.81 (751) | 71.56 (760) |
| Risky/high risk drinkers | 4.87 (525) | 6.80 (64) | 8.00 (85) |
| Missing | 0.84 (91) | 0.96 (9) | 1.6 (17) |
| BMI |  |  |  |
| Underweight/acceptable weight | 50.61 (5455) | 46.65 (439) | 45.48 (483) |
| Overweight/Obese | 44.98 (4848) | 49.95 (470) | 47.74 (507) |
| Missing | 4.42 (476) | 3.4 (32) | 6.78 (72) |
| OC use |  |  |  |
| No | 93.48 (10076) | 92.56 (871) | 93.88 (997) |
| Yes | 6.27 (676) | 7.23 (68) | 5.74 (61) |
| Missing | 0.25 (27) | 0.21 (2) | 0.38 (4) |
| HRT use |  |  |  |
| No | 81.42 (8776) | 79.91 (752) | 74.48 (791) |
| Yes | 18.34 (1977) | 19.55 (184) | 25.52 (271) |
| Missing | 0.24 (26) | 0.53 (5) |  |

Table S3: The Hazard ratio and 95% confidence interval of reproductive life variables and breast cancer incidence among Australian women who born 1946-1951

| Reproductive life factors | uHR (95% CI) | aHR (95% CI) | uSHR (95% CI) | aSHR (95% CI) |
| --- | --- | --- | --- | --- |
| Smoking Status |  |  |  |  |
| Non-smoker | Ref |  | Ref |  |
| Ex-smoker | 0.95 (0.81-1.10) | 0.96 (0.82-1.12) | 0.94 (0.81-1.10) | 0.96 (0.82-1.12) |
| Current smoker | 1.07 (0.90-1.28) | 1.05 (0.88-1.26) | 1.03 (0.86-1.22) | 1.01 (0.85-1.22) |
| **CP global test** | P=0.32 | P>0.05 |  |  |
| Alcohol consumption |  |  |  |  |
| Non-drinker | Ref |  | Ref |  |
| Rarely drinks /low-risk drinkers | 1.19 (0.98-1.45) | 1.16 (0.95-1.42) | 1.21 (1.00-1.47) | 1.18 (0.97-1.44) |
| Risky/high-risk drinkers | 1.59 (1.17-2.16) | 1.61 (1.19-2.19) | 1.58 (1.16-2.14) | 1.60 (1.18-2.18) |
| **CP global test** | P<0.01 | P<0.05 |  |  |
| BMI |  |  |  |  |
| Underweight/Acceptable weight | Ref |  | Ref |  |
| Overweight/Obese | 1.18 (1.03-1.34) | 1.16 (1.02-1.32) | 1.18 (1.03-1.34) | 1.16 (1.02-1.32) |
| **CP global test** | P<0.05 | P<0.01 |  |  |
| Marital status |  |  |  |  |
| Partnered | Ref |  | Ref |  |
| Non-partnered | 1.30 (1.11-1.52) | 1.33 (1.13-1.57) | 1.27 (1.08-1.49) | 1.31 (1.11-1.54) |
| **CP global test** | P=0.37 | P=0.29 |  |  |
| OC use |  |  |  |  |
| No | Ref |  | Ref |  |
| Yes | 1.17 (0.91-1.49) | 1.15 (0.90-1.48) | 1.16 (0.91-1.49) | 1.15 (0.90-1.48) |
| **CP global test** | P=0.91 | P=0.76 |  |  |
| HRT use |  |  |  |  |
| No | Ref |  | Ref |  |
| Yes | 1.06 (0.9-1.24) | 1.04 (0.88-1.23) | 1.04 (0.88-1.22) | 1.03 (0.87-1.21) |
| **CP global test** | P=0.92 | P=0.75 |  |  |
| Age at first pregnancy |  |  |  |  |
| 11-19 Years | Ref |  | Ref |  |
| 20+ Years | 1.01 (0.86-1.20) | 1.02 (0.85-1.22) | 1.02 (0.86-1.21) | 1.02 (0.85-1.21) |
| Not applicable | 0.90 (0.66-1.22) | 0.78 (0.46-1.32) | 0.88 (0.65-1.20) | 0.77 (0.44-1.333) |
| **CP global test** | p=0.39 | p=0.55 |  |  |
| Age at menarche |  |  |  |  |
| <10 Years | Ref |  | Ref |  |
| >10 Years | 0.93 (0.70-1.27) | 1.46 (0.58-3.71) | 0.94 (0.69-1.27) | 1.47 (0.58-3.71) |
| Not applicable | 0.57 (0.23-1.45) | 1.47 (0.61-3.56) | 0.56 (0.22-1.42) | 1.48 (0.61-3.56) |
| **CP global test** | p=0.67 | p=0.74 |  |  |
| Parity |  |  |  |  |
| None | Ref |  | Ref |  |
| One | 1.29 (0.90-1.84) | 1.11 (0.67-1.83) | 1.29 (0.90-1.85) | 1.11 (0.66-1.86) |
| Two or more | 1.08 (0.81-1.42) | 0.92 (0.57-1.49) | 1.10 (0.83-1.46) | 0.93 (0.56-1.54) |
| **CP global test** | p=0.41 | p=0.25 |  |  |

Adjusted for age, income, ariacity, marital status, education, country of birth, alcohol, BMI, smoking, OC use, HRT use, age at first full-time pregnancy, age at menarche, and parity

uHR=Unadjusted hazard ratio, aHR=Adjusted hazard ratio, uSHR=unadjusted sub-distribution hazard ratio, aSHR=Adjusted sub-distribution hazard ratio, CI= Confidence interval
