# Supplementary material for: Influence of lifestyle factors on breast cancer incidence from mid-life to older age: an Australian longitudinal cohort study
Source: BMJ Open. 2026 Mar 29;16(3):e105193. doi: 10.1136/bmjopen-2025-105193 (PMC13034360; doi:10.1136/bmjopen-2025-105193)
Supplement: Supplementary file 2 [file bmjopen-2025-105193.reviewer_comments.pdf]

PEER REVIEW HISTORY

BMJ Open publishes all reviews undertaken for accepted manuscripts. Reviewers are asked to complete a checklist review form and are provided with free text boxes to elaborate on their assessment. These free text comments are reproduced below.

ARTICLE DETAILS

Title (Provisional)

Influence of lifestyle factors on breast cancer incidence from mid-life to older age: An Australian longitudinal cohort study

Authors

Rana, Md Sohel; Marinovich, Luke; Houssami, Nehmat; Cavenagh, Dominic; Byles, Julie E; Rahman, Md Mijanur; Yu, Xue Qin

---

VERSION 1 - REVIEW

**Reviewer**	**1**
**Name**	**Katapodi, Maria C.**
**Affiliation**	**University of Basel, Clinical Research**
**Date**	**20-Jul-2025**
**COI**	**None**

The authors describe associations of BMI, levels of alchohol consumption, smoking, and other known risk factors with the incidence of breast cancer among a sample of approximately 12,000 Australian women that were followed prospectively for 23 years. The prospective, longitudinal study design and the large sample are the major strengths of the study.

The comments below will strengthen the paper:

1) overall, editing for English language is needed - use of articles, use of tenses, etc.

2) Page 5, line 11: "Deterministic methods were used for linking ..." Please, define "deterministic methods" or edit

3) Define how you separated "low risk drinking" from "high risk drinking". Please provide as exact measures as possible, to allow replication and comparison to other studies.

4) Did the study account for level of smoking, e.g., number of cigarettes per day, pack-years, etc.? If not, acknowledge the in the limitations.

5) Consider providing the exact questions asked to assess self-reported health behaviors, even if this information has been reported in previous publications

6) The study did not account for first pregnancy after age 30, which is a major risk factor for breast cancer according to the Gail model. Please, acknowledge this in the limitations

7) Please explain "non-applicable" for age of menarche

8) Provide a reference for Schoenfeld statistical test

9) Table 1 and Tables in supplementary material: move % (n) to the line of Education, as the heading does not apply for age (mean, SD)

**Reviewer**	**2**
**Name**	**Hammer, Marilyn**
**Affiliation**	**Dana-Farber Cancer Institute, Nursing and Patient Care Services; Medical Oncology, Division of Population Sciences**
**Date**	**29-Oct-2025**
**COI**	**None**

The premise of this study was to evaluate factors that increased the risk for breast cancer from mid-life to older adult age among women who were enrolled in the ALSWH study and had data linked to the Australian Cancer Database. This is an important study and novel approach. Addressing a few areas would strengthen this manuscript, including:

1. Please correct the reporting of BMI categories. Should be <18.0 = underweight; 18.0-24.9 = normal weight; >=25.0 – 29.9 = overweight; >= 30.0 obese

2. On page 5, the sentence, “The majority were born in Australia (75.34% versus 75.77%)” is difficult to interpret. Please clarify.

3. In the methods section under analysis, please include that the Cox Proportional Hazards Model was used to evaluate hazard ratios.

4. Analyzing BMI as a continuous variable may have provided better insights regarding BMI and BC risk. In addition, evaluating interaction effects would have been informative. It is recommended that these analyses be conducted or include in the limitations that they were not conducted.

**Reviewer**	**3**
**Name**	**Huang, Li-Ching**
**Affiliation**	**Vanderbilt University, Biostatistics**
**Date**	**18-Dec-2025**
**COI**	**None**

---

1. The authors state that participants were followed until 2022 (page 3, lines 39–40); however, they also indicate that the censoring date was set at the end of 2019 (page 5, lines 19–20; page 4, line 5). Please clarify this discrepancy.

2. Should the censoring date be defined as the date of last contact, corresponding to the date of the final completed survey?

3. How were missing values in the covariates for the multivariable regression model handled?

4. Please report the follow-up time separately for participants with BC and for those without BC.

5. Please remove the sentence on page 5, lines 9–10 (‘Differences between groups…’), as no statistical tests (Chi-square or t-test) were conducted to support this statement.

VERSION 1 - AUTHOR RESPONSE

**Reviewer 1**	**Author responses**	**Changes in the track-changed manuscript (text shown in italic)**
The authors describe associations of BMI, levels of alcohol consumption, smoking, and other known risk factors with the incidence of breast cancer among a sample of approximately 12,000 Australian women that were followed prospectively for 23 years. The prospective, longitudinal study design and the large sample are the major strengths of the study.<br><br>The comments below will strengthen the paper:	We appreciate your efforts in reviewing this manuscript and believe that your comments and suggestions will help us improve the paper.	

1) Overall, editing for English language is needed - use of articles, use of tenses, etc.	Thanks for this comment. We have thoroughly checked the language and updated it accordingly.	Please see the changes in the "Main document-marked copy"
2) Page 5, line 11: "Deterministic methods were used for linking ..." Please, define "deterministic methods" or edit	We appreciate your comment. We have defined deterministic methods in the manuscripts and provided citations.	Definition of deterministic methods of data linkage is provided in the manuscript (lines 124-128 on page 4):<br>*A deterministic linkage based on agreement of common unique identifiers was used for linking cancer information for the ALSWH participants from January 1982 onwards across all Australian states and territories. Details on the linked cancer data are documented elsewhere [26, 27].*<br>Newly added reference *(lines 441-442 on page 15)*<br>*Carreras, G., et al., Deterministic and Probabilistic Record Linkage: an Application to Primary Care Data. Journal of Medical Systems, 2018.* ***42****(5): p. 82*
3) Define how you separated "low risk drinking" from "high risk drinking". Please provide as exact measures as possible, to allow replication	In the Australian Longitudinal Study on Women Health (ALSWH), alcohol consumption status was defined according to the National Health and Medical Research Council	We added the following definition in the 'Methods' section under 'Exposures' sub-section of the manuscript (lines 139-142 on page 4-5):

and comparison to other studies.	(NHMRC) alcohol guidelines. The NHMRC alcohol guidelines align with the WHO alcohol guidelines, with the overarching goal of reducing alcohol related harm.<br><br>Based on the amount and pattern of drinking, risk levels are as follows:<br><br>**Non-drinker:** No alcohol consumption.<br><br>**Rarely drinks / low-risk drinkers:** Drinking within the guideline threshold for reducing lifetime harm. No more than 10 standard drinks per week and no more than 4 on any day.<br><br>**Risky / high-risk drinkers:** Drinking above the guideline threshold for lifetime harm (e.g., >10 drinks/week or >4 on a single day).	*According to the National Health and Medical research Council (NHMRC) guidelines, which align with the WHO guidelines, rarely/low-risk drinkers were defined as those who consume ≤10 standard drinks per week and ≤4 on any single day, while risky/high-risk drinkers were those who exceed these limits.*
4) Did the study account for level of smoking, e.g., number of cigarettes per day, pack-years, etc.? If not, acknowledge the in the limitations.	No, we did not include the level of smoking in this study. We have acknowledged the issue as a limitation according to your suggestion.	We added the following limitation in the manuscript (lines 327-328 on page 12):<br><br>*We did not account for the level of smoking (e.g., number of cigarettes per day, pack-years) due to inconsistency across survey waves….*

5) Consider providing the exact questions asked to assess self-reported health behaviours, even if this information has been reported in previous publications	The questions related to self-reported health behaviours are publicly available in the ALSWH websites. As requested, we have now provided the exact questions for alcohol consumption and smoking in the questionnaires to the manuscript.	We have added exact questions for alcohol consumption and smoking in the 'Methods' section under 'Exposure' sub-section (lines 136-147 on page 4-5):<br>*Data on alcohol consumption was collected based on the questions "How often do you usually drink alcohol?" and "On a day when you drink alcohol, how many standard drinks do you usually have?". According to the National Health and Medical research Council (NHMRC) guidelines, which align with the WHO guidelines, rarely/low-risk drinkers were defined as those who consume <=10 standard drinks per week and <=4 on any single day, while risky/high-risk drinkers were those who exceeded these limits. Smoking status was characterised based on the questions "How often do you currently smoke cigarettes or any tobacco products?" and "In your lifetime, would you have smoked at least 100 cigarettes (or equivalent)?". The ALSWH derived smoking status variable based on responses from these two questions and labelled*

		(non-smoker, ex-smoker, current smoker)<br><br>We also have given reference for readers on all aspects of the ALSWH including questionnaire in the 'Methods' section under the sub-section 'Data source and sample' (lines 107-109 on page 4)<br><br>*Details about the ALSWH survey design, recruitment methods, questionnaires, response rates have been published elsewhere [23, 25] and are also available at www.alswh.org.au.*
6) The study did not account for first pregnancy after age 30, which is a major risk factor for breast cancer according to the Gail model. Please, acknowledge this in the limitations	Thanks for this comment. We agree	We have added the following sentence in the limitation section (lines 327-329 on page 12):<br><br>*We did not account for the level of smoking (e.g., number of cigarettes per day, pack-years) due to inconsistency across survey waves and first pregnancy after age 30 as it is not asked at the baseline survey.*
7) Please explain "non-applicable" for age of menarche	In the ALSWH study, "Not applicable" was mentioned when a participant never had a menstrual period. This might happen due to congenital	We changed in the manuscript follows as (lines 160-162 on page 5):

	reproductive anomalies, genetic conditions (e.g., turner syndrome), or endocrine disorders.	*Reproductive variables were parity (none, one, and two or more); age at first full-time pregnancy (11-19 years, 20+ years, and not applicable); and age at menarche (<10 years, >10 years, and not applicable [participant never had a menstrual period].*
8) Provide a reference for Schoenfeld statistical test	We have added the reference.	Here is the newly added reference (lines 451-452 on page 15):<br>*Schoenfeld, D., Partial residuals for the proportional hazards regression model. Biometrika, 1982.* ***69****(1): p. 239-241.*
9) Table 1 and Tables in supplementary material: move % (n) to the line of Education, as the heading does not apply for age (mean, SD)	We agree and have updated accordingly.	Please refer to the "Main document-marked copy"
**Reviewer 2**		
1. Please correct the reporting of BMI categories. Should be <18.0 = underweight; 18.0-24.9 = normal weight; >=25.0 – 29.9 = overweight; >= 30.0 obese	We dichotomized BMI (<25 kg/m^2=underweight/acceptable weight, and >25 kg/m^2=overweight/obese), because the prevalence of underweight and obese is comparatively small.	We justified BMI categorization in 'Methods' section under 'Exposures' sub-section (lines 130-135 on page 4):<br>*BMI measured from self-reported height and weight (<18 kg/m^2=underweight, ≥18 to <25 kg/m^2=acceptable weight, ≥25 to <30*

		kg/m²=overweight, ≥30 kg/m²=obese). Since the prevalence of underweight and obese is comparatively small, we collapsed BMI into two categories (<25 kg/m²=underweight/acceptable weight, and ≥25 kg/m²=overweight/obese).
2. On page 5, the sentence, "The majority were born in Australia (75.34% versus 75.77%)" is difficult to interpret. Please clarify.	We have modified the sentence based on the reviewer's feedback.	Modified sentence is as follows (lines 205-207 on page 6):<br>*Women with BC and those without were similar in terms of country of birth, with over three-quarters (75.34% versus 75.77%) born in Australia.*
3. In the methods section under analysis, please include that the Cox Proportional Hazards Model was used to evaluate hazard ratios.	We appreciate your comment. We have added the following sentence in the methods under analysis section.	The suggested addition in 'Methods' section under 'Statistical analysis' is reflecting in the following (lines 187-188 on page 6):<br>*A Cox proportional hazards model was also used to evaluate hazard ratios and 95% CI (reported in Supplementary Table S3).*
4. Analyzing BMI as a continuous variable may have provided better insights regarding BMI and BC risk. In addition, evaluating interaction effects would have been informative. It is	Thanks for pointing out two important points in this comment. We agree with your argument; however, we used BMI as a categorical variable in our analysis for plausible interpretation.	We added the following point as limitation in the last paragraph of discussion (lines 325-327 on page 12):<br>*Potential interaction effects were not assessed in our study*

recommended that these analyses be conducted or include in the limitations that they were not conducted.		*due to the complexity of modelling time-varying interactions, which limits the ability to determine whether the observed associations differed across subgroups.*
**Reviewer 3**		
Comments to the Author:	Thanks for your valuable feedback on our manuscript. We have addressed your comments number by number in the following:	
1. The authors state that participants were followed until 2022 (page 3, lines 39–40); however, they also indicate that the censoring date was set at the end of 2019 (page 5, lines 19–20; page 4, line 5). Please clarify this discrepancy.	Thank you for identifying this issue. The ALSWH survey data are available through 2022 (10th survey), whereas cancer data from the ACD are available through 2019 (the most recent available for linkage). That is why we censored at the end of 2019.	For more clarification we added the following justification in the 'Methods' section under the 'Data source and sample' sub-section (lines 101-103 on page 3):<br>*However, only follow-up data until 2019 (ninth follow-up) was used, given that the ACD data were not available beyond this year.*
2. Should the censoring date be defined as the date of last contact, corresponding to the date of the final completed survey?	Censoring was defined as 31st December 2019, rather than the date of last survey contact. Complete cancer ascertainment, including exact diagnosis dates, is available through registry linkage for the entire follow-up period for all participants (irrespective of survey return status).	

	Therefore, loss to survey follow-up does not affect cancer outcome ascertainment.	
3. How were missing values in the covariates for the multivariable regression model handled?	We used a missing indicator for variables with missing values for the multivariable regression model.	Our manuscript consists of the following paragraph on missing value handling in analyses (lines 166-170 on page 5):<br>*Missing values for these variables were filled in if available in the nearest adjacent surveys. However, a small proportion (<5%) of the socio-demographic variables were not filled as there was no available data in adjacent surveys. Complete case analysis was used in all models after filling missing data from adjacent surveys.*
4. Please report the follow-up time separately for participants with BC and for those without BC	We agree and have reported persons-years of follow-up time in the second row of Table 1	Please see the changes in the second row of Table 1.
5. Please remove the sentence on page 5, lines 9–10 (‘Differences between groups…’), as no statistical tests (Chi-square or t-test) were conducted to support this statement.	Thank you. We have removed the sentence accordingly.	The change has been made in the “Main document-marked copy” for your consideration.

VERSION 2 - REVIEW

**Reviewer**	**2**
**Name**	**Hammer, Marilyn**
**Affiliation**	**Dana-Farber Cancer Institute, Nursing and Patient Care Services; Medical Oncology, Division of Population Sciences**
**Date**	**10-Feb-2026**
**COI**	

The authors adequately addressed the reviewers' comments.

**Reviewer**	**3**
**Name**	**Huang, Li-Ching**
**Affiliation**	**Vanderbilt University, Biostatistics**
**Date**	**23-Feb-2026**
**COI**	

The authors have responded to all my questions. I have no further concerns that require another revision.
